# Null Mutant *mig-15(udn323)* Shows Touch Receptor Neuron Migration Defects in *C. elegans*

**DOI:** 10.17912/micropub.biology.001904

**Published:** 2025-11-25

**Authors:** Sara DaCunha, Gary A Silverman, Tim Schedl, Stephen C Pak, Sara M Fielder, Gervette M Penny

**Affiliations:** 1 Pediatrics, Washington University in St. Louis, St Louis, Missouri, United States; 2 Genetics, Washington University in St. Louis, St Louis, Missouri, United States

## Abstract

The *C. elegans mig-15* gene is an essential driver of Q-neuroblast polarization and migration. *mig-15* mutations alter the migration of Q-neuroblast descendants. However, the effects of complete loss-of-function of *mig-15* on *C. elegans* Touch Receptor Neuron (TRN) migration are not well-characterized. Therefore, we used CRISPR/Cas9 to delete the entire *mig-15* gene and assessed the migration of AVM, ALML, ALMR, and PVM TRNs using fluorescence microscopy. In the *mig-15 (udn323)* null mutant, AVM and PVM neurons often fail to migrate to their correct positions. These findings show that complete loss of *mig-15* significantly alters the migration of *C. elegans* TRNs.

**Figure 1. AVM and PVM touch receptor neurons display migration defects in mig-15 knockout mutants:**
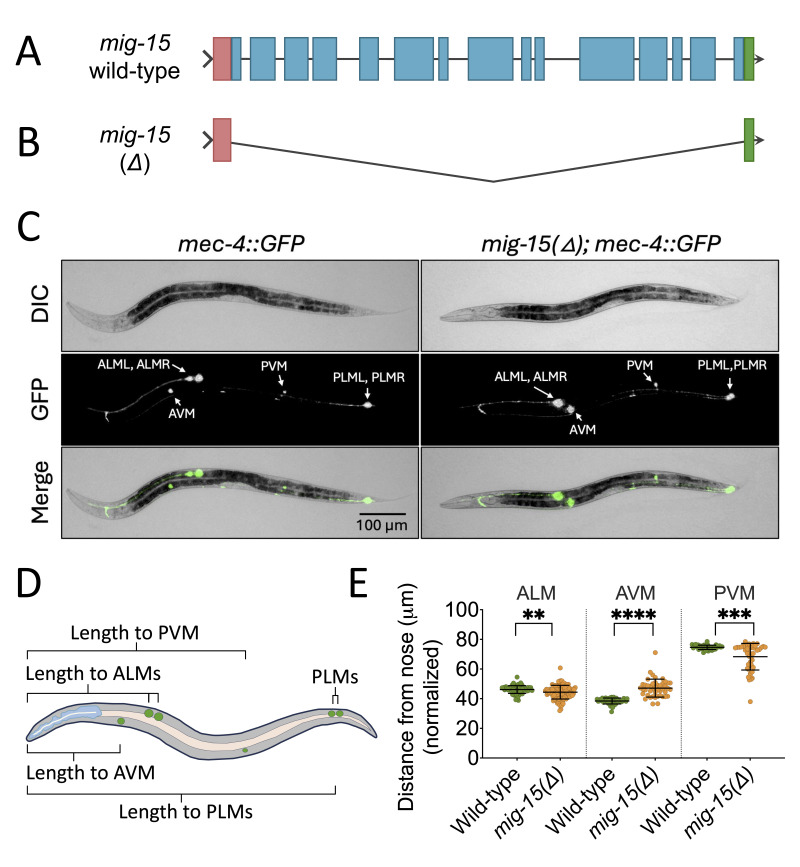
**(A)** Schematic diagram of the *mig-15* gene that contains 15 exons. The 5'- and 3'- UTR regions are shown in pink and green, respectively. The exons are shown in blue. The introns are shown as black lines. (B) The *mig-15 (Δ)* animals lack all 15 exons of the *mig-15* gene. **(C)** Representative DIC (top), GFP (middle), and merge (bottom) images of wild-type (left) and *mig-15 (Δ)* (right) animals expressing *mec-4::GFP* . In this *mig-15 (Δ)* image, the AVM shows abnormal migration past the ALMs, while the PVM is positioned normally. **(D)** Schematic diagram of the TRN measurements employed. Measurements for the AVM, the ALMs, and the PVM were taken from the tip of the nose cone to the center of each neuron. Body length measurement was taken from the tip of the nose cone to the center of the farthest migrated PLM. All measurements were made using Fiji (ImageJ Version 2.16) and normalized to the total length of the animal. **(E)** Quantification of ALM (left; WT: n=86, D: n=92; note, there are 2 ALMs in each animal), AVM (middle; WT: n=43, *D* : n=45), and PVM (right; WT: n=42, *D* : n=46) migration in L4 hermaphrodite animals. Statistically significant differences in TRN migration were observed for all three neuron types. While ALMs showed a mild migration defect, the AVM and PVM showed a more severe migration defect. Statistical analysis performed using a Mann-Whitney Ranks test in GraphPad Prism Version 10.6.1.

## Description

*mig-15* is an essential gene in *C. elegans* that encodes for a Nck-interacting kinase (NIK) (Poinat et al. 2002). It is required for cell fate, polarization, and migration of Q-neuroblasts and their descendants (Chapman et al. 2008). The Q neuroblasts are the precursor cells that give rise to both mechanosensory and touch receptor neurons (TRNs). The AVM and PVM TRNs are formed post-embryonically (Sulston and Horvitz 1977), and arise from two Q-neuroblast precursor cells, the QR (AVM) and the QL (PVM). Both the QR and the QL cells are born at similar posterior-lateral positions and later migrate either anteriorly (QR) or posteriorly (QL) to form their respective descendant cells (Chapman et al. 2008). The related AQR (located in the anterior region of the animal) and PQR neurons (located in the posterior region of the animal) are also formed from the QR and QL cells, respectively. *mig-15* was shown to be important for PQR posterior migration through WNT signaling activated *mab-5* Hox expression (Chapman et al. 2008). The six non-ciliated TRNs extend long neurites across the animal's body to regulate movement in response to touch. The TRNs include the Anterior Lateral Microtubule Left (ALML) cell, the Anterior Lateral Microtubule Right (ALMR) cell, the Anterior Ventral Microtubule (AVM) cell, the Posterior Lateral Microtubule Left (PLML) cell, the Posterior Lateral Microtubule Right (PLMR) cell, and the Posterior Ventral Microtubule (PVM) cell (Sundararajan and Lundquist 2012). The ALMs and PLMs, which are formed embryonically (Syntichaki and Tavernarakis 2004), are in the anterior (head) and posterior (tail) regions of the animal, respectively (Zhang et al. 2013).

Previous studies have shown that *mig-15 ( rh148 )* (missense) and *mig-15 ( rh80 )* (stop-gain) mutants show defects in the ability of Q-neuroblasts to polarize, maintain polarization, and migrate. The mutant Q-neuroblast cells were also frequently observed to only migrate partially before dividing. These mutations alter the migration patterns of the AQR and PQR neurons (Chapman et al. 2008; Shakir et al. 2006). Mutants in *mig-21 ,* shown to be a crucial factor in initial migration of QR and QL cells, also exhibit abnormal AQR and PQR migration (Du and Chalfie 2001). *mig-15* mutants additionally affect migration and axonal outgrowth of touch receptor neurons. For example, a *mig-15 ( mu342 )* strong loss-of-function mutant showed PLM termination hook defects and PLM branch defects. Animals that lacked a PVM cell body were also observed in the *mig-15 ( mu342 ); rpm-1* double mutant (Crawley et al. 2017). *mig-15* was also found to be important for ventral axon outgrowth in AVM and PVM TRNs (Teulière et al. 2011). These studies highlight the importance of *mig-15* in mechanosensory and PLM touch receptor neuron development. However, very little is known about how *mig-15* mutations specifically affect migration of TRNs. To study the effects of complete loss of *mig-15* on migration of the TRNs, we used CRISPR/Cas9 to delete the entire *mig-15* gene, from start codon to stop codon.

The *mig-15* gene contains 15 exons **( Figure 1A )** . To generate the *mig-15 (udn323)* deletion strain (hereafter referred to as *mig-15 (Δ)* ), two guide RNAs, located in the first and last exons ( **Figure 1B** ), and a repair template for homologous recombination containing 5'UTR sequence up to the start codon and 3'UTR sequences up to the stop codon were employed. To maintain the *mig-15 (Δ)* animals, a balancer chromosome ( *tmC24* ) was introduced. Homozygous *mig-15 (Δ)* adult animals from deletion heterozygous hermaphrodites ( *mig-15 (Δ)/ tmC24 )* are maternally rescued (m+). These adult animals are small, sickly, and display Egl (egg-laying defective), Unc (uncoordinated), and Dpy (dumpy) phenotypes. We were unable to assay *mig-15 (Δ)* ; *mec-4::GFP* (m-) animals from the next generation since these animals produce very few progeny; they are sickly and severely developmentally delayed. The *mig-15 (Δ)* null allele phenotype closely resembles the previously described *mig-15 ( rh326 )* early stop-gain allele (Shakir et al. 2006). This observation indicates that *mig-15 ( rh326 )* is likely a null allele. Using a full-gene deletion null allele for this study will allow us to more thoroughly assess the effects of *mig-15* on TRN migration, as opposed to other alleles.

To observe migration of TRNs, we crossed the *mig-15 (Δ)/ tmC24* with the *mec-4::GFP* ( *zdIs5* ) marker that labels the TRNs (ALM, AVM, PVM, PLM). We then collected *mig-15 (Δ)* (m+) homozygotes and used fluorescence microscopy to image the TRNs and measure their final migration distances compared to wild-type. As shown in **Figure 1C** , abnormalities in TRN migration were observed in the *mig-15 (Δ)* animals. The most striking migration defects were observed in the AVM and PVM neurons. Most AVM neurons failed to complete their migration towards the head and were incorrectly distributed along the anterior of the animal. Several AVMs migrated posteriorly, and we observed one AVM closer to the region where PVMs are located. Interestingly, the PVM neurons exhibit a bimodal phenotype. Approximately half of the PVM neurons exhibited abnormal anterior migration patterns characteristic of wild-type AVMs, while the remainder differentiated near their original birthplace as expected. To quantify the severity of these migration defects, we measured the distance of each neuron from the nose of the animal **( Figure 1D )** . Each neuron type was identified for measurement based on its ventral or dorsal position, branch shape, and/or direction of neuron process extension. A statistically significant difference in AVM and PVM migration was observed for *mig-15 (Δ)* compared to wild-type. One animal was identified where the AVM cell body was completely missing. We also observed a small, but significant, difference in ALM migration the *mig-15 (Δ)* **( Figure 1E )** .

Our visual observation was that PLM migration was not affected. AVM and PVM migration defects were not reported in the *mig-15 ( mu342 )* strain, and it is possible that this mutation does not affect the function of *mig-15* responsible for TRN migration, or this was not assessed. The bimodal migration distribution observed with PVMs in the *mig-15 (Δ)* is similar to that previously observed in *mig-15* mutants with QL and QR neuroblasts during their initial polarization and migration, with about half of them migrating to their correct positions over the V5 and V4 seam cells, respectively (Chapman et al. 2008). In the *mig-15 ( rh148 )* and *mig-15 ( rh80 )* mutants, a subset of the PQR mutants migrate normally, and others terminate prematurely. Studies have shown that the PVM also responds to WNT - *mab-5* signaling, which is important for posterior migration of Q-neuroblast descendants (Siddiqui and Culotti 1991). Therefore, it is likely that our observations result from a lack of migration cues through *mig-15- mab-5* signaling, which results in these cells making a random decision.

This is the first study to use a *mig-15* full-gene deletion to study TRN migration in *C. elegans* . We present the finding that a *mig-15* null mutation significantly alters the migration patterns of AVM and PVM TRNs. Taken together, this work adds to the existing body of knowledge that *mig-15* is important for migration of both TRN and mechanosensory neurons in *C. elegans* .

## Methods

To generate the *mig-15 (Δ)* strain, we injected *C. elegans* wild-type VC2010 one-day-old adults with Cas-9, tracrRNA, crRNA, and pRF4 *rol-6* plasmid (co-transformation injection marker), as previously described (Boulin et al. 2021; Huang et al. 2022). To remove the entire gene, we used two guide RNAs, located in the first and last exons of *mig-15 ,* ( **Figure 1B** ), and a repair template for homologous recombination containing flanking 5'UTR sequence up to the start codon and 3'UTR sequence up to the stop codon. PCR product from primers flanking the deleted region was sequenced to confirm that *udn323* *mig-15* coding sequence was deleted from start to stop codon. The strain was outcrossed two times to VC2010 before use. The homozygous *mig-15 (Δ)* adults produce very few progeny before death, so a balancer chromosome with a red pharynx marker ( *tmC24* ) was used to maintain *mig-15 (Δ)* as a heterozygote. The fluorescent marker *mec-4::GFP* labels TRNs (AVM, ALM, PVM, PLM). To cross the *mec-4::GFP* marker into the *mig-15 (Δ)* / *tmC24* line, a two-step scheme was used. *mig-15* is on the X-chromosome, and uncoordinated (Unc) *mig-15* ( *Δ)* hemizygous males and *tmC24* hemizygous males cannot mate. Therefore, *mec-4::GFP* males were crossed into strain FX30253 ( *tmC24 [ F23D12.4 (tmIs1233) unc-9 (tm9718)] X; tmEx4950* ). This line is homozygous for *tmC24* and contains an extrachromosomal array with wild-type *unc-9* and a GFP intestinal marker. The males from this cross were then used to cross into the *mig-15 (Δ)* / *tmC24* line so that the balancer would be retained.

To assess migration of TRNs, 10 L4 hermaphrodite animals were picked and placed into 5 µL of 50 mM sodium azide to anesthetize the worms atop an agarose (2% in H _2_ O) pad. Differential Image Contrast (DIC) and fluorescence images were captured using a 10x objective on a Zeiss Axioplan 2 compound fluorescent microscope. DIC and fluorescent images were then uploaded to ImageJ for analysis. The “segmented line” feature was used to trace the length of the animal, from the tip of the nose cone to the center of the PLMs. Measurements were also taken at the center of the AVM, ALML, ALMR, and PVM in the TRN experiments. Numerical data were normalized by dividing each neuron measurement by the length of the animal to account for slight differences in body size.

## Reagents

**Table:** 

**Strain**	**Allele**	**Full Genotype**	**Available from**

VC2010	Wild-type	n/a	CGC
UDN100659	*mig-15 (udn323) *	*mig-15 (udn323) / tmC24* [ F23D12.4 *(tmIs1240)* *unc-9 (tm9719)* ] (X)	This work
FX30194	*tmC24*	*tmC24* [ F23D12.4 *(tmIs1240)* *unc-9 (tm9719)* ] (X)	CGC
UDN100673	*mig-15 (udn323) / tmC24* (X); *zdls5* (I)	*mig-15 (udn323)* / *tmC24* [ F23D12.4 *(tmIs1240)* *unc-9 (tm9719)* ] (X); *zdls5* [ *mec-4p::GFP* + *lin-15 (+)* ] (I)	This work
SK4005	*zdIs5*	*zdIs5* [ *mec-4 p::GFP* + *lin-15 (+)* ] (I)	CGC
FX30253	*tmEx4950*	*tmC24* [ *myo-2p::mCherry* , : F23D12.4 ] (X). *tmEx4950* [ *unc-9 (+)* + *vha-6p::GFP* ]	CGC

**CRISPR Reagents**	**Sequence/description**		**Available from**

*mig-15* gRNA1 (in 5' coding)	CCTGATGACGACATGGTTTT		IDT
*mig-15* gRNA2 (in 3' coding)	GGGTTGACAAATTGGTAAAC		IDT
*mig-15* ( *udn323)* repair template	Gtgtggtttaagtgtcggcttgctccaagccgctcacagcacccaaaaccactggtctcgaatcaatcgttttccattttttaaaatatgtatttgtaa		IDT
Cas9	Alt-R™ S.p. HiFi Cas9 Nuclease V3 ( IDT 108060)		IDT
pRF4 (pCCM958)	*rol-6 ( su1006 ).* Contains the *rol-6* collagen gene as an extrachromosomal array. Used as a co-transformation marker. Generates rollers. Not transgenerational.		n/a
tracrRNA	Alt-R™ CRISPR-Cas9 tracrRNA (IDT 1072533)		IDT
crRNA	Alt-R™ CRISPR-Cas9 crRNA with targeting sequence TGCAGATCAAGTGTACGACG		IDT

**Genotyping Primers**	**Sequence**		**Available from**

*mig-15 (udn323)* F1	CGCCAGCACAGGTATGATAA		IDT
*mig-15* ( *udn323)* R1	CCTGGATGGATGCTTGAAAT		IDT
mig-15 ( *udn323)* F2	TTTCCCGGCTGTGTAACAAT		IDT
